# New plastomes of eight *Ipomoea* species and four putative hybrids from Eastern Amazon

**DOI:** 10.1371/journal.pone.0265449

**Published:** 2022-03-17

**Authors:** Marcele Laux, Renato R. M. Oliveira, Santelmo Vasconcelos, Eder S. Pires, Talvâne G. L. Lima, Mayara Pastore, Gisele L. Nunes, Ronnie Alves, Guilherme Oliveira

**Affiliations:** 1 Instituto Tecnológico Vale, Belém, Pará, Brazil; 2 Programa Interunidades de Pós-Graduação em Bioinformática, Universidade Federal de Minas Gerais, Belo Horizonte, Minas Gerais, Brazil; 3 Programa de Pós-Graduação em Botânica Tropical, Museu Paraense Emílio Goeldi, Belém, Pará, Brazil; Chinese Academy of Medical Sciences and Peking Union Medical College, CHINA

## Abstract

*Ipomoea* is a large pantropical genus globally distributed, which importance goes beyond the economic value as food resources or ornamental crops. This highly diverse genus has been the focus of a great number of studies, enriching the plant genomics knowledge, and challenging the plant evolution models. In the Carajás mountain range, located in Eastern Amazon, the savannah-like ferruginous ecosystem known as *canga* harbors highly specialized plant and animal populations, and *Ipomoea* is substantially representative in such restrictive habitat. Thus, to provide genetic data and insights into whole plastome phylogenetic relationships among key *Ipomoea* species from Eastern Amazon with little to none previously available data, we present the complete plastome sequences of twelve lineages of the genus, including the *canga* microendemic *I*. *cavalcantei*, the closely related *I*. *marabaensis*, and their putative hybrids. The twelve plastomes presented similar gene content as most publicly available *Ipomoea* plastomes, although the putative hybrids were correctly placed as closely related to the two parental species. The *cavalcantei-marabaensis* group was consistently grouped between phylogenetic methods. The closer relationship of the *I*. *carnea* plastome with the *cavalcantei-marabaensis* group, as well as the branch formed by *I*. *quamoclit*, *I*. *asarifolia* and *I*. *maurandioides*, were probably a consequence of insufficient taxonomic representativity, instead of true genetic closeness, reinforcing the importance of new plastome assemblies to resolve inconsistencies and boost statistical confidence, especially the case for South American clades of *Ipomoea*. The search for k-mers presenting high dispersion among the frequency distributions pointed to highly variable coding and intergenic regions, which may potentially contribute to the genetic diversity observed at species level. Our results contribute to the resolution of uncertain clades within *Ipomoea* and future phylogenomic studies, bringing unprecedented results to *Ipomoea* species with restricted distribution, such as *I*. *cavalcantei*.

## Introduction

Located in Eastern Amazon, the Carajás mountain range harbors the altitude ferruginous savannah-like ecosystem known as *canga*, characterized by shallow soils (0–10 cm) and potentially phytotoxic levels of metals [1]. The *canga* plateaus are isolated from each other by matrixes of rainforest and show high levels of both endemism and species turnover as the result of environmental heterogeneity [1–3]. Previous studies have found evidence that the *canga* soil properties are restrictive for the seedling establishment, working as primary drivers of vegetation composition and structure in the ecosystem [4–6]. Several studies have been performed with plant and animal populations sampled in the Carajás National Forest, or Floresta Nacional de Carajás, progressively improving the knowledge about the biodiversity and genetic diversity patterns from native and endemic populations, as well as to investigate the occurrence of hybridization and speciation events [2, 5–11].

*Ipomoea* species are broadly distributed in the world, especially in the tropics and subtropics [12–14], where about 600–700 species are known [12], or about 800–900 species in the broader generic concept based on recent phylogenetic analysis, considering the genera *Argyreia*, *Astripomoea*, *Blinkworthia*, *Lepistemon*, *Lepistemonopsis*, *Mina*, *Paralepistemon*, *Rivea*, *Stictocardia* and *Turbina* nested within *Ipomoea* [11, 15, 16]. The genus *Ipomoea* is recognized within the tribe Ipomoeeae, which has been subdivided into two main clades [11, 16, 17], with long history of taxonomic and nomenclatural problems [16, 18]. Widely known as “morning glories” or “bindweeds”, the species of the genus present a high commercial value, either as ornamental plants or food crops [13, 19], with the sweet potato (*I*. *batatas* (L.) Lam.) being one of the most widely cultivated species [20–22]. This genus has also served as a model for understanding many evolutionary questions and elucidate inter and intra-species relationships among populations [6, 9, 15, 18, 22–29].

**Fig 1:**
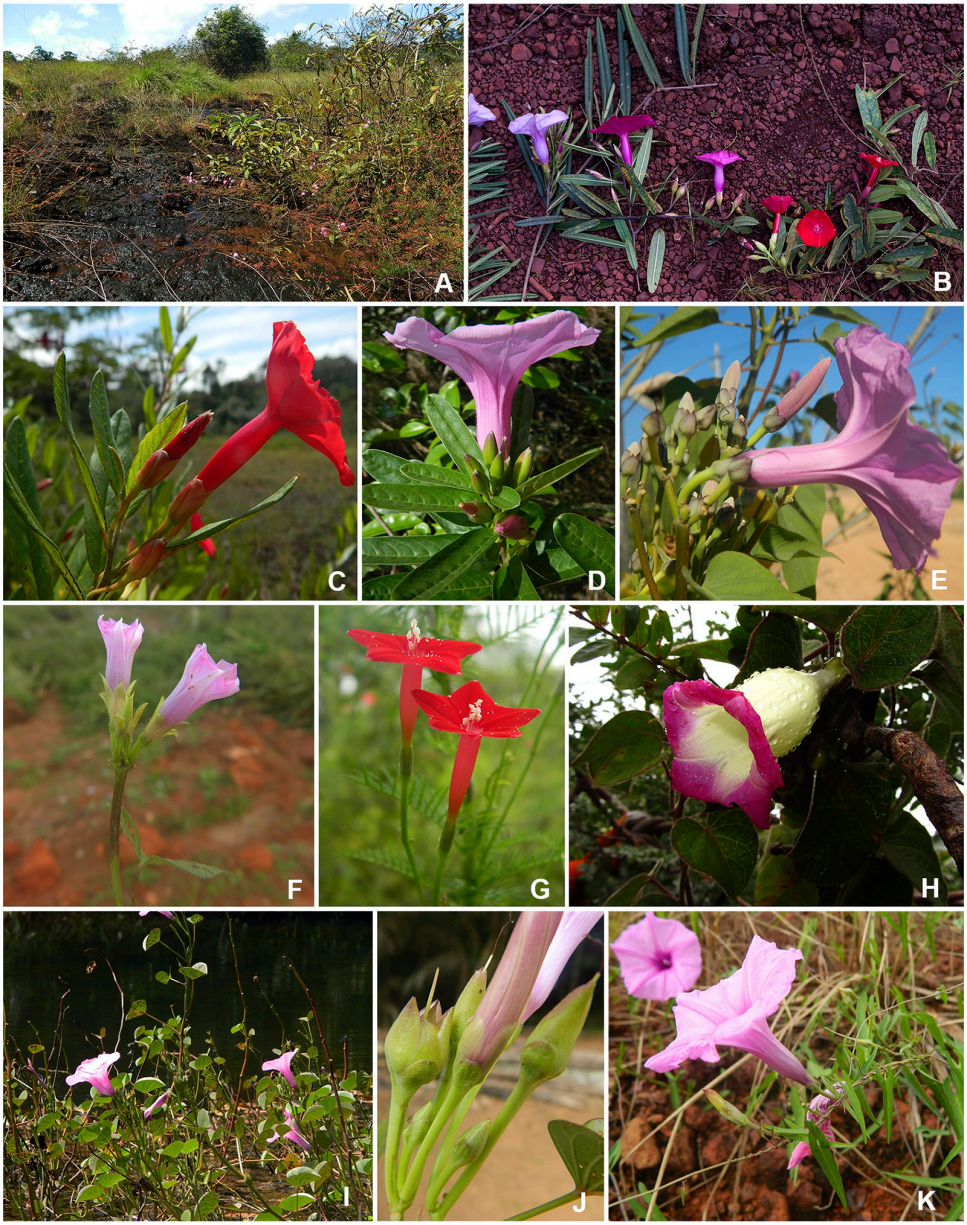
Diversity of *Ipomea* species and putative hybrids analyzed in this work. The canga environment (**A**); *Ipomoea cavalcantei* × *I*. *marabaensis* putative hybrids (**B**); *I*. *cavalcantei* (**C**); *I*. *marabaensis* (**D**); *I*. *carnea* (**E**); *I*. *triloba* (**F**); *I*. *quamoclit* (**G**); *I*. *goyazensis* (**H**); *I*. *asarifolia* (**I** and **J**); and *I*. *maurandioides* (**K**). Photos by Mayara Pastore (A, C-G, I-K), Pedro L. Viana (B) and Marcos E. L. Lima (H).

The most recent inventory of the flora of the *cangas* of Carajás presented 116 seed plant families, encompassing 856 species [30]. Convolvulaceae is represented by eight genera and 20 species in Carajás, 12 of which belonging to the genus *Ipomoea* (Fig 1), including the flagship species *I*. *cavalcantei* D.F. Austin, known as “flor de Carajás” (flower of Carajás), endemic to the ferruginous fields from Carajás North ridge (N1 to N5) and considered an endangered species [30, 31]. *Ipomoea marabaensis* D.F. Austin & Secco is found in several ferruginous and granitic fields from Carajás, as well as in other rock outcrops in the states of Pará and Tocantins [2, 31]. *Ipomoea cavalcantei* and *I*. *marabaensis* are sister species, with similar genome sizes, shared chloroplast polymorphisms and overlaps in gene allele distributions [5]. The two species occur in sympatry in the *canga* sites N4 and N5, however, *I*. *cavalcantei* is common in N4, whereas *I*. *marabaensis* appears as small groups closer to the *canga*-forest boundaries [6]. Both species are clambering shrubs, perennials, and present elliptical, oblong to obovate leaves, being remarkably similar in terms of vegetative traits. They are mostly differentiated by the flower morphology, with a hypocrateriform deep red corolla adapted to hummingbird pollination in *I*. *cavalcantei*, and a campanulate to infundibuliform light pink to lilac corolla adapted to bee pollination in *I*. *marabaensis* [6, 12, 31, 32] (Fig 1C and 1D). Recently, a preliminary phylogenetic analysis was published, using a concatenated alignment of seven chloroplast genes and positioning *I*. *cavalcantei* and *I*. *marabaensis* within the Murucoides clade [5].

Considering that that the modern plant classification systems [33] relies heavily on molecular data, which are often of chloroplast origin, the plastid genomes are efficient data source for building phylogenies on a broad scale and set species boundaries, inter-population variation, and gene flow at a local scale [34–38]. Also, recent studies have shown the higher variability in phylogenetic signal and different rates of evolution throughout the plastid regions and genes, as previously expected by single locus interpretations [39–44]. Therefore, the assembly of complete plastomes have been extremely useful for providing an abundance of additional characters that can be used to resolve polytomies in phylogenetic trees [18, 45–48] and boost statistical confidence in deeply branching clades [49, 50].

As several individuals with intermediate phenotypes between *I*. *cavalcantei* and *I*. *marabaensis* have been observed at the N4 plateau (Table 1; Fig 1B), where the geographic ranges of both species overlap [5, 6, 31], we aimed to describe and analyze the complete plastome sequences from *I*. *cavalcantei*, *I*. *marabaensis* and their putative hybrids, plus six other *Ipomoea* species from Eastern Amazon, in order to better understand the phylogenetic relationships among the lineages of the genus in the region, besides providing genetic information to direct conservation planning in the unique *cangas* of the Serra de Carajás.

**Table 1:** List of *Ipomoea* lineages studied in the present work, including sampling information and plastome sequencing data for each analyzed specimen.

Species	Sample number	Collection information	GenBank accession	Sequenced reads	Mapped reads	Plastome size (bp)	Mean coverage
*I*. *asarifolia*	ITV3285	E. Babiytchuk, s.n.; Salinópolis, Pará, Brazil	MK086048	45,993,456	807,204	160,589	760.2
*I*. *carnea*	ITV2324	E. Babiytchuk, s.n.; Salinópolis, Pará, Brazil	MK086049	71,273,986	2,241,650	160,819	2125.4
*I*. *cavalcantei*	ITV3206	E. Babiytchuk, s.n.; N1, Serra Norte, Parauapebas, Pará, Brazil	MK086050	517,935,995	745,649	161,563	714.8
*I*. *goyazensis*	ITV4320	L.V. Vasconcelos, 1116; N4, Serra Norte, Parauapebas, Pará, Brazil	MK086051	55,417,682	243,786	160,414	229.8
*I*. *marabaensis*	ITV2328	E. Babiytchuk, s.n.; Serra do Tarzan, Canaã dos Carajás, Pará, Brazil	MK086052	410,313,272	465,351	161,324	440.2
*I*. *maurandioides*	ITV4245	E. Babiytchuk, s.n.; Serra Norte, Parauapebas, Pará, Brazil	MK086053	29,932,159	501,367	161,242	472.7
*I*. *quamoclit*	ITV4995	M.G.C Nogueira, 665; Redenção, Pará, Brazil	MK086054	83,709,896	1,248,317	160,836	1175.4
*I*. *triloba*	ITV4963	M.G.C. Nogueira, 651; Redenção, Pará, Brazil	MK086056	37,827,247	830,975	161,835	774.4
*I*. *cavalcantei* × *I*. *marabaensis* H1	ITV2181	R.M. Harley, 57491; N4, Serra Norte, Parauapebas, Pará, Brazil	MK086044	55,410,366	798,743	161,495	754.6
*I*. *cavalcantei* × *I*. *marabaensis* H2	ITV280	F. Santos, E2FLO.05; N4, Serra Norte, Parauapebas, Pará, Brazil	MK086047	59,399,262	946,578	160,765	899.9
*I*. *cavalcantei* × *I*. *marabaensis* H3	ITV2294	E. Babiytchuk, s.n.; N4, Serra Norte, Parauapebas, Pará, Brazil	MK086045	65,210,419	551,682	160,974	527.5
*I*. *cavalcantei* × *I*. *marabaensis* H4	ITV2295	E. Babiytchuk, s.n.; N4, Serra Norte, Parauapebas, Pará, Brazil	MK086046	65,302,820	750,030	161,948	709.0

## Materials and methods

### DNA extraction and sequencing

DNA extractions were carried out following the automated protocol previously described [51], with approximately 20 mg of plant material collected in NaCl-saturated CTAB solution [52]. Afterwards, paired-end libraries were constructed from 50 ng of DNA. Samples were subjected to a step of enzymatic and random fragmentation in which the DNA were simultaneously fragmented and bound to adapters using the QXT SureSelect kit (Agilent Technologies) according to the manufacturer’s instructions. The fragmented DNA was purified and subjected to an amplification reaction using primers complementary to the adapters. Next, the libraries were quantified using the Qubit 3.0 (Invitrogen) fluorimeter and checked for fragments size in the 2100 Bioanalyzer (Agilent Technologies). Then, the libraries were diluted in a solution of 0.1% Tris-HCl and Tween and pooled. The sequencing run was performed with a NextSeq 500 v2 kit high-output (300 cycles).

### Plastome assembly and annotation

The quality of the generated dataset was checked using the FastQC 0.11.5 tool [53], and the adapters were removed using Trimmomatic 0.38 [54]. The assembly was performed using a hybrid strategy with the de novo NOVOPlasty (NP) v2.6.3 assembler [55] and selected contigs from SPAdes v3.11 [56]. The NP config file was set as follows: insert size 300, read length 150, type chloro, genome range 120k-200k, K-mer 39, paired-end mode and original dataset with the full content of the DNA extracted as input. The seeds initially used for contig extension in NP assembler were genes from the reference genome *I*. *nil* (L.) Roth [57], and subsequently genes from the assembled plastomes, in a recursive strategy. The main seeds used for capture and contig extension were (in order of effectiveness) the complete sequences of the genes psbK, psbB, psaC, ndhF, rbcL, psbC, rpoB, rrn23, trnH, ycf1, rps15, matK, rpl32, and some partial sequences, as the junctions between the two inverted repeat (IR) regions and the small single copy (SSC) region, and the ycf1-rps15 intergenic spacer. The resulting contigs were assembled in Geneious R11 (Biomatters) and the consensus sequences were annotated in the CpGAVAS web server [58].

SPAdes assembler is not designed to deal with the chloroplast (cp) genome architecture, especially because the inverted repeats, so the larger contigs generated were about 27–89 kbp long. To select chloroplast contigs in the SPAdes assembly, the contigs were chosen according to a two-step selection. The chloroplast DNA fragments are expected to be more abundant in the total DNA extracted, since such organelle is found in high numbers within each plant cell [59, 60]. A coverage cutoff was applied according to the overall contigs depth of coverage (DP) median. The selected high DP contigs were subsequently aligned to the plastid NCBI database (ftp://ftp.ncbI.nlm.nih.gov/refseq/release/plastid/) using MegaBLAST [61] with an e-value of 1e-5 and minimum percent identity of 85. The chloroplast contigs sequences were then extracted from the original SPAdes output.

Most plastomes were assembled only with NP, but for manual intervention and gap filling, we used the selected chloroplast contigs from SPAdes. For each sample, after the first draft genome was entirely assembled and annotated in CpGAVAS, the IRs were extracted and pairwise aligned. The NP and SPAdes selected contigs were mapped against the draft genome, and the contigs which fell in such regions were used to guide the sequence edition. The final draft genome was then checked and edited using Artemis v18 [62] for curation. The twelve original datasets were re-mapped against the final plastomes using Bowtie2 [63] to check for coverage uniformity throughout the entire plastome. The duplicates were removed, and the mapped reads were used to calculate the average coverage for each assembly. The variable regions were located using the Geneious Find Variation tool, adopting *I*. *cavalcantei* as the reference genome for the population in study, since the four putative hybrids were closely related to it. RepeatMasker [64] was used to identify and locate the di- to pentameric and some hexameric simple repeat sequences with more than 20 bp, using default parameters. The circular map was generated using OGDRAW [65].

### Phylogenetic analysis of concatenated genes

Thirteen complete genes from the 12 *Ipomoea* plastomes assembled, plus seven *Ipomoea* references (*I*. *nil*, *I*. *purpurea* (L.) Roth, *I*. *trifida* (Kunth) G.Don, *I*. *batatas*, *I*. *hederacea* Jacq, *I*. *lacunosa* L., *I*. *triloba* L.), and one outgroup (*Solanum dulcamara* (Moench) Dunal) were aligned using MAFFT v7 [66] multiple aligner using default settings, and concatenated using Geneious R11. The concatenated alignment of 13 genes selected according to interspecies variability, molecular function, chloroplast compartments, and conservation level (atpA 1524 bp, ccsA 978 bp, matK 1536 bp, ndhB 2222 bp, ndhD 1539 bp, ndhG 531 bp, petA 963 bp, psaA 2253 bp, psaC 246 bp, psbD 1062 bp, rbcL 1443 bp, rpoB 3213 bp and rps15 273 bp) was the input for the phylogenetic inference under the maximum likelihood (ML) criteria with RAxML v8.2.8 [67], using the rapid bootstrapping (1,000 replications) and search for the best-scoring ML tree.

### K-mer frequency distribution

The *Ipomoea* plastomes were also investigated according to the k-mer frequency distribution using the AAF (Alignment and Assembly-Free) software [68] and custom scripts available in S1 Data. AAF reconstructs phylogeny from a distance matrix based on the proportion of shared k-mers between each sample. The k-mer lengths of 25, 31 and 35 were compared according to the support values and congruence with the ML tree generated with the concatenated genes matrix, and the k-mer length of 31 was adopted. The k-mers presenting frequencies with the highest dispersion values (sd function) among the samples were selected and extracted using a R custom script, aligned to consensus sequences and the plastome regions where the high dispersion k-mers were identified were referred as H-disp regions, potentially representing highly variable plastome sites, showing significantly distinct frequency patterns. Using the dispersion among the frequencies as a genetic diversity value parameter, we were able to progressively reduce the number of k-mers, selecting those with the highest contribution.

## Results

### Plastome assembly and comparative analysis

The plastomes were deposited in GenBank under the accession numbers MK086044-MK086056 (Table 1). All datasets presented high quality on average and high mean coverage, according to the respective assembled plastomes (Table 1). A total of 2,693 NP contigs and 11,442 SPAdes contigs were used to perform the assemblies of the 12 specimens. SPAdes generated more contigs, but a few consensus sequences by dataset.

**Fig 2:**
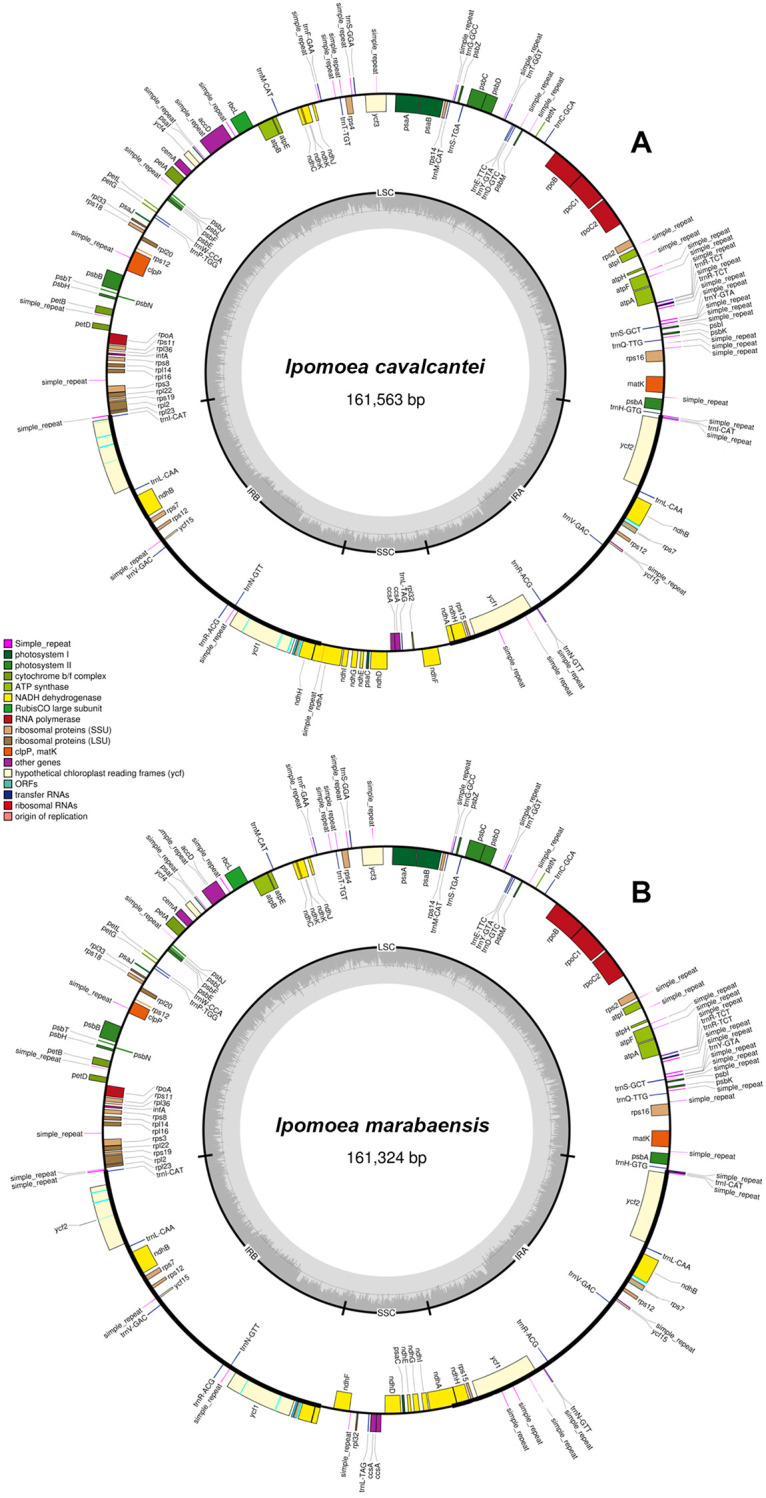
Circular map of the plastomes of *Ipomoea cavalcantei* (A) and *I*. *marabaensis* (B), showing the typical quadripartite structure indicated in the inner circle (IRB, LSC, IRA and SSC). The gene categories are shown in colored boxes and simple repeats sites are indicated in pink.

**Fig 3:**
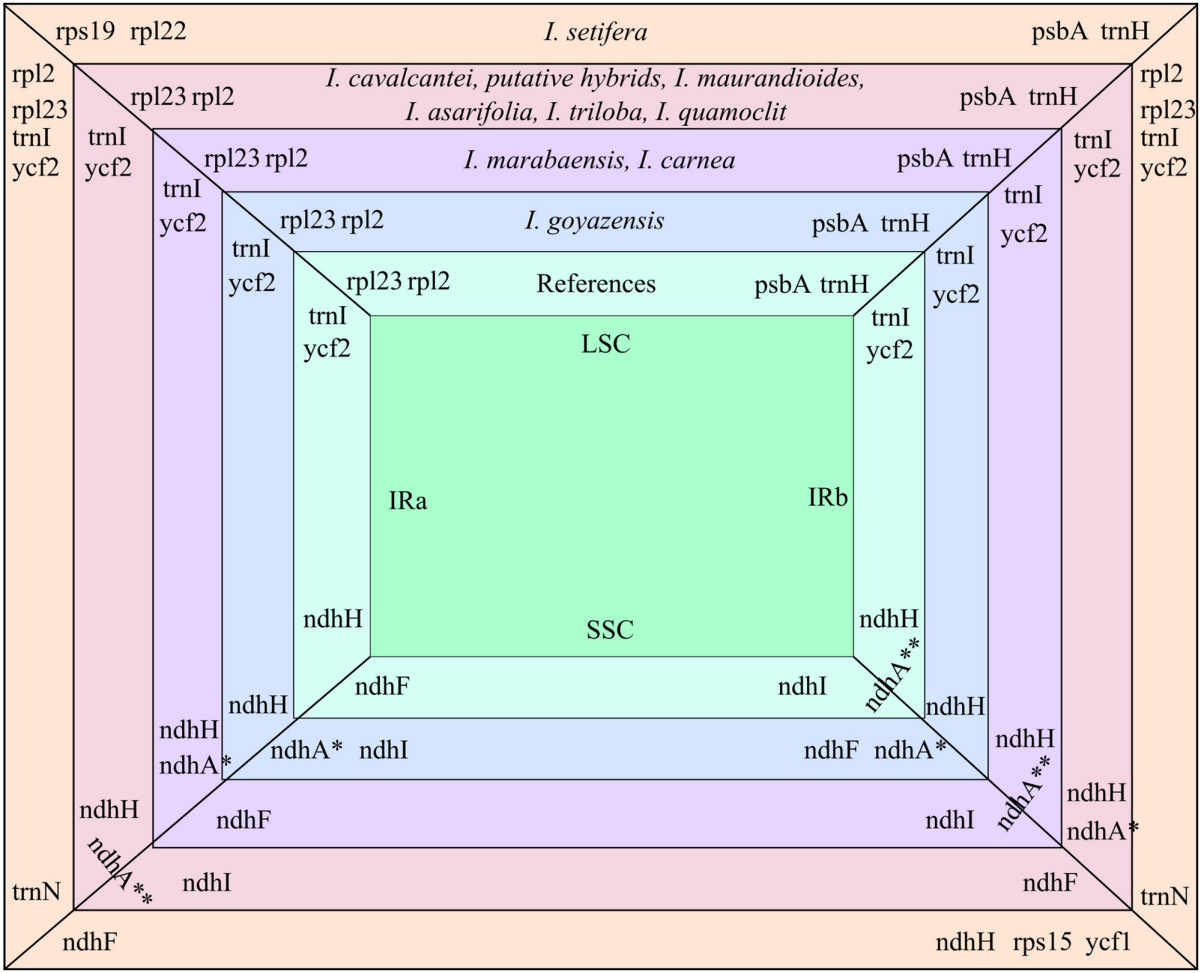
Comparison of the four junctions of the chloroplast quadripartite structure, grouping species with similar junctions, based on [21]. *ndhA with one exon; **ndhA with two exons.

The average length of the chloroplast genomes was 161 kbp, presenting a total of 123 genes, including 80 protein coding genes, one partial infA gene, four rRNA genes, and 33 tRNA genes, with nine tRNA genes presenting at least one duplicate (Table 2). The 12 plastomes assembled displayed the usual circular quadripartite structure (Fig 2), including one large single copy (LSC, 87 kbp in average), one SSC (12 kbp in average), and two IRs (IRa and IRb, 30 kbp in average). The average GC-content of the LSC region was 36%, and 40.8% in the IRs. Most plastomes presented a similar genome structure, with slight rearrangements in the SSC regions (Fig 3). *Ipomoea marabaensis* (Fig 2B) and *I*. *carnea* Jacq. presented the same SSC gene order as the seven *Ipomoea* references, displaying the complete ndhA gene (two exons) in SSC-IRb junction, while for remaining assembled plastomes the complete copy of the ndhA gene was located in the IRa-SSC junction (Fig 2A). All plastomes showed two complete copies of the ycf1 located within the inverted repeats, close to each SSC junction.

**Table 2:** Gene content in the assembled plastome of *Ipomoea cavalcantei*.

Cell function	Gene codification	Observed genes
Self-replication	DNA-directed RNA polymerase	rpoA, rpoB, rpoC1 and rpoC2
Self-replication	Large subunit ribosomal protein	rpl2, rpl14, rpl16, rpl20, rpl22, rpl23, rpl32, rpl33 and rpl36
Self-replication	rRNA genes	rrn4.5^r^, rrn5i^r^, rrn16^r^ and rrn23^r^
Self-replication	Small subunit ribosomal protein	rps2, rps3, rps4, rps7^r^, rps8, rps11, rps12^r^^,^^s^, rps14, rps15^r^, rps16, rps18 and rps19
Self-replication	tRNA genes	trnA^i^^,^^r^, trnC, trnD, trnE, trnF, trnGt, trnH, trnI^d^^,^^i^^,^^r^, trnL^i^^,^^r^^,^^t^, trnK^i^, trnM^d^, trnN^r^, trnP, trnQ, trnR^r^^,^^t^, trnS^t^, trnT^d^, trnV^d^^,^^i^^,^^r^, trnW and trnY^d^
Photosynthesis	ATP synthase	atpA, atpB, atpE, atpF, atpH and atpI
Photosynthesis	Cytochrome b6/f complex	petA, petB, petD, petG, petL and petN
Photosynthesis	NADH oxidoreductase	ndhA^p^^,^^r^, ndhB^r^, ndhC, ndhD, ndhE, ndhF, ndhG, ndhH^R^, ndhI, ndhJ and ndhK
Photosynthesis	Photosystem I	psaA, psaB, psaC, psaI and psaJ
Photosynthesis	Photosystem II	psbA, psbB, psbC, psbD, psbE, psbF, psbH, psbI, psbJ, psbK, psbL, psbM, psbN, psbT and psbZ
Photosynthesis	Rubisco	rbcL
Other functions	Acetyl-CoA-carboxylase subunit	accD
Other functions	c-type cytochrome synthesis	ccsA
Other functions	Envelop membrane protein	cemA
Other functions	Maturase	matK
Other functions	Protease	clpP
Other functions	Translational initiation factor	infA^p^
Putative genes	Conserved ORF	ycf1^r^, ycf2^r^, ycf3, ycf4 and ycf15^r^

^d^duplicated;

^i^intron;

^p^partial;

^r^inverted repeat;

^s^trans-splicing; and

^t^triplicated

Thirty-five and 33 polymorphic simple repeats were identified in *I*. *cavalcantei* and *I*. *marabaensis*, respectively, mostly located in intergenic regions (Fig 2), seven located in the coding regions of the plastomes of *I*. *cavalcantei* (accD, atpF, clpP, ndhA, trnR, trnY and ycf3,) and five of *I*. *marabaensis* (accD, atpF, clpP, trnY and ycf3). All chloroplast genomes presented a homogeneous coverage throughout the entire plastome and presented mean coverage above 200× in the remapping analysis (Table 1). The genes and regions with higher interspecific variability among the plastomes analyzed were accD, clpP, matK, ndhB-rps7, ndhG, psaA, rpoC1, rps16 and ycf2.

The H-disp regions with higher contribution (frequency distribution dispersion) were the ycf1 repeat, also found in the reference genomes, at the same position, and the H-disp regions found in rps15 gene, in the intergenic region between ndhH and rps15, ycf2 and trnL and between rps7 and ndhB genes. The H-disp between rps7 and ndhB was not present in *I*. *goyazensis*. The H-disp found in the ycf1 gene consisted in a 48 bp region which occurred in two copies, except for *I*. *carnea*, which contained only a single copy (Table 3). We found the ycf1 H-disp repeat in the reference genomes, even for *I*. *batatas*, which lacks the ycf1 gene [21] but presents the two copies of the repeat in the same position, between trnN and rps15.

**Table 3:** H-disp regions captured through the k-mer frequency dispersion among the samples.

Plastome region	H-disp
5’-ndhB	TTCATTCTGTACATGCCAGCTCATGAATTAGT
ndhH-rps15	CTTATTTGTTTCGTTTCAATTTTGA
rps7-ndhB	TTCCAATTTCAAAAAAAAATCCCAATTGTGT
rps15	GTACGTTATAAAGAATTAATTGAGAAATTGGATATTCGAGAGACAAA
trnI-ycf2	GTTTTCAAGTAATGTTTGATCAATTACGTATTTATACACGTATTCGTATTAATCAATTTTTGATGAATTA
ycf1 repeat	GAAGAGATCAATCCCAGCAGTAATCAAAAGACTCCAATTGGGACTAAT

### Phylogenetic reconstruction

Both tree reconstruction methods showed high support for the *cavalcantei-marabaensis* group and *I*. *carnea* was the closest related species among the analyzed specimens, also grouping *I*. *asarifolia* and *I*. *maurandioides*, with *I*. *quamoclit* being placed in the same branch. Both methods also showed the distinct relationship between the putative hybrids and the two sister species.

**Fig 4:**
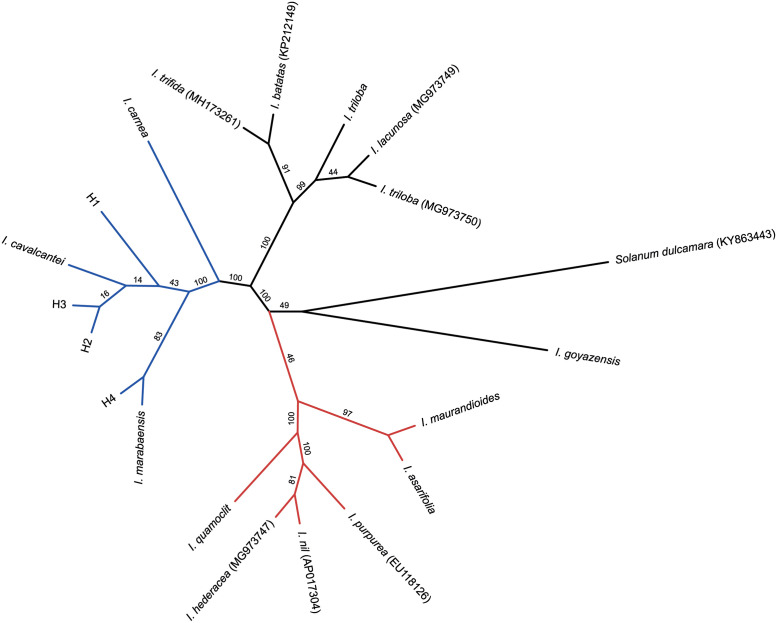
Phylogenetic relationships among *Ipomoea* species. Unrooted ML phylogenetic tree of the 12 *Ipomoea* sequenced plastomes, plus five *Ipomoea* plastomes previously available in public databases and one outgroup from Solanales (*Solanum dulcamara*) using RAxML with the GTR+G model and rapid bootstrap with 1,000 replicates, based on a concatenated matrix of 13 genes. The *cavalcantei-marabaensis* group is highlighted in blue and the *quamoclit-asarifolia-maurandioides* in brown.

**Fig 5:**
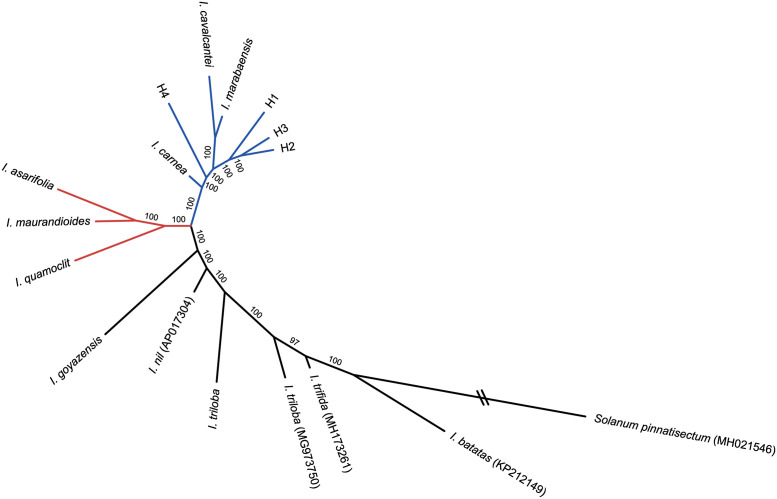
Dendrogram representing the genetic distances among *Ipomoea* species. Unrooted genetic distance tree based on k-mer frequency distribution using the AAF approach, using the 12 *Ipomoea* sequenced plastomes, four *Ipomoea* plastomes previously available in public databases and one outgroup from Solanales (*Solanum pinnatisectum*). The *cavalcantei-marabaensis* group is highlighted in blue and the *quamoclit-asarifolia-maurandioides* in brown.

The putative hybrids H1, H2 and H3 were closely related to *I*. *cavalcantei*, while H4 showed a higher similarity to the *I*. *marabaensis* plastome, according to the ML tree (Fig 4). On the other hand, the two parental plastomes were closely related to each other according to the AAF tree, and the H4 appeared as sister to the remaining lineages of the *cavalcantei-marabaensis* group (Fig 5). Three larger groups were formed according to the ML trees, being the *cavalcantei-marabaensis* group (BS = 100), the weakly supported *asarifolia-maurandioides-quamoclit* group (BS = 49) and the *triloba-batatas* group (BS = 100) (Fig 4). However, among those, only the *cavalcantei-marabaensis* group was recovered, with the other lineages forming different clusters (Fig 5). *Ipomoea asarifolia* and *I*. *maurandioides* were closer between each other in both methods (BS > 97), but the grouping with *I*. *quamoclit* was inconsistent (BS = 46) in the ML analysis, showing *I*. *quamoclit* closer (BS > 81) to three *Ipomoea* references from the Pharbitis clade (*I*. *hederacea*, *I*. *nil* and *I*. *purpurea*). *Ipomoea triloba* was grouped in a highly supported branch with four references, all belonging to the Batatas clade, but showing an inconsistent polyphyletic pattern with a reference of the same species, *I*. *triloba* MG973750 (Figs 4 and 5). Most of the differences between the branches were found in intergenic regions. Among the coding regions, we highlight the accD and clpP genes, which were the most variable genes.

The phylogenetic reconstruction based on the assembly-free shared k-mers (AAF) generated a total of 1,187,671 k-mers for all 12 datasets and two major groups could be observed (Fig 5). Similar to the ML tree, the *cavalcantei-marabaensis* group was highly supported (BS = 100) and *I*. *carnea* was, again, the closest relative. The *asarifolia-maurandioides-quamoclit* branch was not topologically consistent and the long branches reflect the large genetic distance between *I*. *asarifolia* and *I*. *quamoclit* (Fig 5).

## Discussion

### Interspecific genetic diversity

With the advent of the next-generation sequencing, a substantial increase in number of organelle genomes newly assembled has been observed, along with the remarkable expansion of studies investigating new evolutionary and structural diversification patterns, which reinforce the importance of the genome-wide genetic diversity exploration and gene variability, especially below the order and family levels [39, 42, 43]. We identified potentially variable sites according to single nucleotide variants and the H-dips regions, which pointed to genes harboring potentially informative genetic diversity sites. The accD gene was among the most variable genes, as previously observed for *Ipomoea* plastomes [26, 40, 69]. The genes clpP, ndhA, rps16, ycf1 and ycf2 also showed high interspecific variability, especially clpP, which is highly variable and presents a remarkable acceleration in plastome evolutionary rate [44, 69, 70]. Shared chloroplast polymorphisms between *I*. *cavalcantei* and *I*. *marabaensis* in rpoC1 was previously observed [5]. We could not find any reference for the ycf1 H-disp repeat in the repeats databases, but the ycf1 gene is considered a genomic marker [27, 71] and variable sites were also identified by different studies [26, 27, 29, 72]. The H-disp region found between ndhH and rps15 was also observed in all plastomes, and already adopted as marker region for species discrimination in some studies [73, 74]. The accD and ycf3 simple repeats were also observed in recently published *Ipomoea* plastomes [21, 27].

### Phylogenetic considerations

The definition of infrageneric clades in *Ipomoea* is hampered by its extreme evolutionary lability in morphology and the widespread homoplasy among the species, especially in highly diverse tropical ecosystems [13, 15, 18, 75, 76]. *Ipomoea cavalcantei* and *I*. *marabaensis* are sister species, sharing plastome types and alleles distribution, but a greater diversity was already documented in ITS2 alleles in *I*. *cavalcantei*, suggesting different diversification rates between the two species [5]. Despite recognized as two species belonging to the Murucoides clade, *I*. *cavalcantei* and *I*. *marabaensis* were not sampled in previous phylogenetic studies of *Ipomoea*, which included mainly better-known species for understanding the infrageneric classification. The Murucoides clade was further investigated, including *I*. *murucoides* Roem. & Schult. (Southern Mexico to Guatemala) and *I*. *polpha* R.W. Johnson (Australia) [18]. According to the most recent and comprehensive study of *Ipomoea* in Americas, both species were arbitrarily placed within the New Word clade called clade A1, related morphologically to species of a smaller clade designated Arborescens [11].

According to recent studies concerning the organization, composition, and inheritance patterns of the organelle genomes, concatenated datasets may present strong variation of phylogenetic signal across the matrix, resulting in distinct or conflicting topologies [39, 42, 43]. Among the 13 genes composing the concatenated matrix, two were chosen for carrying H-disp regions (ndhB and rps15), two variable genes (ndhG and psaA), traditional markers (matK and rbcL), and seven more conserved genes related to self-replication, photosynthesis, and transmembrane molecular function (atpA, ccsA, ndhD, petA, psaC, psbD and rpoB). Both ML and AAF tree reconstruction methods applied in this work showed high support for the *cavalcantei-marabaensis* group, but the relationships among the internal nodes are still unclear. According to the ML method, the putative hybrid H4 was closer to *I*. *marabaensis* (BS = 83), while H1, H2 and H3 were closer to *I*. *cavalcantei*, but with low support values (Fig 4). The occurrence of hybridization may be one of the reasons for the low support internal branches observed [24, 77, 78], especially considering that an interspecific hybridization between the sister species has already been confirmed, producing fertile offspring [6]. Both methods also placed *I*. *carnea* as the closest related species to the *cavalcantei-marabaensis* group among the analyzed plastomes, as well as *I*. *quamoclit* as the closest species to *I*. *asarifolia* and *I*. *maurandioides* plastomes, an incongruent relationship according to previous phylogenetic studies. Despite the AAF highly supported branch, *asarifolia-maurandioides-quamoclit* was artificially grouped probably due to an insufficient sampling representativity, and the long branches demonstrate the substantial genetic distance between the three species (Fig 5). In the ML trees, the *asarifolia-maurandioides-quamoclit* branch support was low (BS = 46) and *I*. *quamoclit* grouped with three references from Pharbitis clade in a high support internal branch. *Ipomoea asarifolia*, *I*. *maurandioides* and *I*. *quamoclit* are not closely related, belonging to different clades within the genus and were probably grouped due the lack of tropical and South American *Ipomoea* references [15], instead of true genetic closeness or low phylogenetic resolution [24, 77, 79, 80].

### Morphological considerations

The vegetative characters combined with the hypocrateriform red corolla make *I*. *cavalcantei* very distinct, tackling problems about its clustering in the identification key with all species of the genus in the Americas [11]. The members of the *cavalcantei-marabaensis* group showed flowers with a sharp color gradient from lilac, considered an ancestral characteristic, to red, which evolved independently within the tribe Ipomoeeae [18, 23, 81] (Fig 1). Both *I*. *cavalcantei* and *I*. *quamoclit* (Quamoclit clade) present a red hypocrateriform corolla, but the two species are not closely related, illustrating the independent diversification process among our sampled *Ipomoea* species plastomes [11, 12, 23]. Moreover, the same SSC gene order observed in *I*. *marabaensis*, *I*. *carnea* and the references, and the higher similarity of H4 putative hybrid with *I*. *marabaensis*, could be related to the heterogeneity in diversification rates [28], the relaxed radiation of Central-South America *Ipomoea* clades [15] or even to the color flower and pollination preferences [23].

The closeness of *I*. *carnea* and the sister species agrees with morphological studies because these species have the woody branches, subequal sericeous sepals, and seeds with long side trichomes. According to [82], *I*. *carnea* belongs to the series Jalapae, while [11] classified it within the sister clade Jalapa. *Ipomoea goyazensis*, the most genetically distant among the plastomes covered in this study (Figs 4 and 5), belongs to a different monophyletic group with about 30 species characterized by subequal coriaceous sepals, glabrous, usually convex and glabrous corolla [11]. The polyphyletic pattern observed for *I*. *triloba* and the same species reference plastome (Figs 4 and 5) is probably a result of low phylogenetic resolution captured by the plastomes or even due to biogeographic genetic variability. On the other hand, the whole branch grouped the described plastome of *I*. *triloba* with the references *I*. *batatas*, *I*. *trifida*, *I*. *triloba* MG973750 and *I*. *lacunosa*, all belonging to the section *Batatas*, confirmed as monophyletic group [11, 15, 18].

Furthermore, the complex plastome relationships observed within the *cavalcantei-marabaensis* group and among the eight sampled species may suggest that our newly assembled plastomes could be from species of a highly diverse South American *Ipomoea* clade, as already proposed, which phylogenetic relationships are currently poorly resolved [11, 15, 22, 25]. In S1 Table we present results of the allele distribution among five chloroplast genes, which show the distinct allele distribution among *cavalcantei-marabaensis* group and the remaining species. In the attempt to define natural clades, phylogenetic inferences were initially proposed and phylogenetic analyses have been testing the monophyly of traditional groups, providing advances towards a biogeographically and taxonomically representative phylogenetic classification of the tribe Ipomoeeae [11, 13–15, 17, 18, 76, 81–85]. However, it is important to emphasize our sampling here was focused on species within a definite geographic scale for conservation purposes. Therefore, the phylogenetic relationships among *I*. *cavalcantei*, *I*. *marabaensis* and the South American *Ipomoea* clade could only be robustly investigated by the inclusion of additional morphologically similar species in future phylogenetic reconstructions, therefore improving the taxonomic resolution with a more comprehensive genetic diversity within the systematic context.

## Conclusions

As research efforts are starting to address the genetic diversity of plant species with restricted geographic distribution in the highly explored ironstone outcrops of the Serra dos Carajás [e.g. 5, 6, 9, 86, 87], the main focus has been directed to understand population dynamics of a few lineages employing NGS-derived SNP analyses. However, there is still the need for a better coverage of genetic studies on the several known endemics of the *cangas* [2]. Yet, phylogenetic diversity analyses using proper cladistic approaches and robust molecular data with high genomic coverage are not being developed in the same pace for the species of the region, with just a couple of previous studies available, including two rare *canga* quillworts [88, 89]. Thus, the results we presented here for *Ipomoea* species are important to provide a better view of the phylogenetic context of rare morning glories, especially considering the occurrence of hybridization and introgression between *I*. *cavalcantei* and *I*. *marabaensis* [6], two important components of the flora of the *cangas*, besides populating the genetic databases with information on one of the most diverse and economically important angiosperm genera.

## Supporting information

S1 Table Visual representation of the allele distributions detected for the genes matK, ndhG, psaA, rpoC1 and rps16. (XLSX)

S1 Data H-disp regions script and analysis pipeline. (PDF)
