# Supplementary material for: New plastomes of eight Ipomoea species and four putative hybrids from Eastern Amazon
Source: PLoS One. 2022 Mar 17;17(3):e0265449. doi: 10.1371/journal.pone.0265449 (PMC8929602; doi:10.1371/journal.pone.0265449)
Supplement: S1 Data — (PDF) [file pone.0265449.s002.pdf]

Supplementary File 1
H-disp regions script and analysis pipeline

###
KmerFilter.R

```
## auxiliar ##
##randomSample = function(df,n) {
##  return (df[sample(nrow(df), n),])
##}

######################################
##
## PCA + high dispersion based kmers
## Marcele Laux & Ronnie Alves
## marcelelaux@gmail.com
##
## 23/05/2019
##
######################################


# SEETING WORKING DIRECTORY (having the AAF generated file)
setwd("C:/Users/DIR/")
# phylokmer.dat: AAF output
#read.table("phylokmer.dat", header=TRUE, skip=15) # skip 15 header
#-k 25
#-n 10
#sample1: ITV1706_P_wullschlaegelii
#sample2: ITV2181_I_cavalcantei_magenta
#sample3: ITV2294ts_I_hybrid
#sample4: ITV2295_I_hybrid
#sample5: ITV2324_I_carnea
#sample6: ITV2328_I_marabaensis
#sample7: ITV2613_I_setifera
#sample8: ITV280_I_hybrid
#sample9: ITV3037_P_carajasense
#sample10: ITV3206_I_cavalcantei
#sample11: ITV3285_I_asarifolia
#sample12: ITV4245_I_maurandioides
#sample13: ITV4320_I_goyasensis
#sample14: ITV4963_I_triloba
#sample15: ITV4995_I_quamoclit

# CREATE DATA FRAME
dset <- read.table("phylokmer.dat", header=TRUE, skip=15) # skip 15
header
#dim(dset)
dset_kmer <- dset[,1] # create a vector having all kmers
dset <- dset[,-1]
# SETTING COLNAMES FOR THE SPECIES IN AFF
colnames(dset) <- c("P_wullschlaegelii","H1","H3","H4",

"I_carnea","I_marabaensis","I_setifera","H2","P_carajasense",

"I_cavalcantei","I_asarifolia","I_maurandioides","I_goyasensis","I_trilob
a",
                    "I_quamoclit")
```

```
#########################################
## LOAD LIBRARIES FOR PLOTTING
library(ggplot2)
library(ggjoy)
library(dplyr)
library(reshape2)
set.seed(1234)
#########################################

df <- melt(dset)
#########################################
##
## HISTOGRAMS OF KMERS DISTRIBUTION TO ALL SPECIES
ggplot(df,aes(x=value)) +
  scale_x_continuous(expand=c(0.02,0),limits=c(40,710),
                     breaks=seq(0,700,100),
                     labels=c("","",200,"",400,"",700,"")) +
  geom_histogram(aes(y=..ncount..),binwidth=5,fill="gray40") +
  facet_wrap(~variable,nrow=4,dir="v") +
  labs(x="kmerFreq",y="plants") +
  theme_bw() +
  theme(axis.text.y=element_blank())

#########################################################
##
## CALCULATING KMERS DISPERSION

dset_t <- data.frame(t(dset),check.names=F) # TRANSPOSE

kmers_sd <- sapply(dset_t,sd) # dispersion
# Ploting histogram of kmer dispersion
hist(kmers_sd)
#plot(density(kmers_sd))

################################################
## SORTING KMERS MATRIX ACCORDING TO DISPERSION
kmers_sd_o <- dset[order(kmers_sd,decreasing = TRUE),]

##############################
## PLOT TOTAL COUNTS OF SPECIES
dotchart(colSums(kmers_sd_o))

#head(order(kmers_sd,decreasing = TRUE))
#head(kmers_sd[order(kmers_sd,decreasing = TRUE)])
#dset[head(order(kmers_sd,decreasing = TRUE)),][10]

## NO ZERO collumns
#kmers_rZero <- rowSums(dset_t == 0)
#index <- rowSums(teste >= 12) # >= 10 or 15

#teste[index,]

#teste[rowSums(teste == 0) <= 4, ]
#teste[apply(teste == 0, 1, sum) <= 4, ]

#save(dset,dset_t,dset_kmer,kmers_sd_o, file = "kmer.RData")
```

```
################################################
##
## FILTERING BY DISPERSION
## GETTING THE TOP-1000 KMERS
##
dsetSmall<-kmers_sd_o[1:1000,]

##############################
## PLOT TOTAL COUNTS OF SPECIES
dotchart(colSums(dsetSmall))

dsetSmall_ts <- t(dsetSmall) # TRANSPOSE FOR HCLUST CLUSTERING

##############################
## PLOT HCLUST TOP-1000 KMERS WITH HIGH DISPERSION
plot(hclust(dist(dsetSmall_ts)))
#group <- cutree(hclust(dist(dsetSmall_ts)), k = 3)

#dsetSmall_ts <- t(dsetSmall)
dsetSmall_df <- as.data.frame(dsetSmall_ts) # DATA FRAME FOR PCA

################################################
##
## FILTERING BY DISPERSION & PCA
##
##
## LOAD LIBRARIES
library("factoextra")
library("FactoMineR")
library("PerformanceAnalytics")
citation("factoextra")
res.pca <- PCA(dsetSmall_df, graph = FALSE) # PCA

fviz_screeplot(res.pca, ncp=10) # SCREE PLOT OF PCA

# BIPLOT OF SPECIES
fviz_pca_ind(res.pca)

#fviz_pca_var(res.pca)

#fviz_pca_var(res.pca, col.var="cos2") +
#  scale_color_gradient2(low="white", mid="blue",
#                        high="red", midpoint=0.5) + theme_minimal()

##########################################
# Contributions of variables on PC1 & PC2
fviz_pca_contrib(res.pca, choice = "var", axes = 1:2)

###################################
# Total contribution on PC1 and PC2
varPC1_PC2 <- fviz_pca_contrib(res.pca, choice = "var", axes = 1:2)
#head(varPC1_PC2$data)

###################################
## PLOT HISTOGRAM OF TOTAL CONTRIBUTION
hist(varPC1_PC2$data$contrib)
```

```
#varPC1_PC2$data[varPC1_PC2$data$contrib>0.11,]
#dim(varPC1_PC2$data[varPC1_PC2$data$contrib>=0.11,])
#rownames(varPC1_PC2$data[varPC1_PC2$data$contrib>0.11,])
#dsetSmall_df[,rownames(varPC1_PC2$data[varPC1_PC2$data$contrib>0.11,])]

########################################
# FILTERING K-MERS THAT CONTRIBUTE MOST
# contribution > 0.11
#
res.pca_filtro <-
dsetSmall_df[,rownames(varPC1_PC2$data[varPC1_PC2$data$contrib>0.11,])]

##############################
## PLOT TOTAL COUNTS OF SPECIES
dotchart(colSums(t(res.pca_filtro)))

##############################
## PLOT HCLUST TOP-1000 KMERS WITH HIGH DISPERSION
plot(hclust(dist(res.pca_filtro)))

##############################
## SAVE ALL VARIABLES
save.image(file = "kmer.RData")

######################
##
## GENERATING KMER LIST
dset_kmer_noFactor <-
as.numeric(as.vector(unlist(colnames(res.pca_filtro))))
dset_kmer[dset_kmer_noFactor]
#######################################
#  WRITING TSV LIST INTO A TSV FILE
#
write.table(as.data.frame(dset_kmer[dset_kmer_noFactor]),
file="filter_kmer_list.tsv",
            row.names = FALSE, col.names = FALSE)

########################################
##
## HEATMAPS
library(gplots)
library(RColorBrewer)
#####################

#heatmap(data.matrix(res.pca_filtro))
#plot(hclust(dist(res.pca_filtro)))

# select indexes for labelling clusters of species
group <- cutree(hclust(dist(res.pca_filtro)), k = 3)
group[group==1] <- "1"
group[group==2] <- "2"
group[group==3] <- "3"

# select indexes for labelling clusters of species
# based on a set of 3 colors
col1 <- brewer.pal(3, "Set3")

# make a heatmap with the final matrix (15x432)
```

```
heatmap.2(data.matrix(res.pca_filtro), col=greenred(75),
          trace="none",
          keysize=1,
          margins=c(6,20),
          scale="none",
          dendrogram="row",
          Colv = TRUE,
          Rowv = TRUE,
          cexRow=0.5 + 1/log10(dim(res.pca_filtro)[1]),
          cexCol=1.25,
          main="kmer-15 15x432",
          RowSideColors= col1[as.numeric(group)]
)

# make a heatmap with a subsample of the final matrix (15x100)
heatmap.2(data.matrix(res.pca_filtro[,1:100]), col=greenred(75),
          trace="none",
          keysize=1,
          margins=c(6,20),
          scale="none",
          dendrogram="row",
          Colv = TRUE,
          Rowv = TRUE,
          cexRow=0.5 + 1/log10(dim(res.pca_filtro)[1]),
          cexCol=1.25,
          main="kmer-15 15x100",
          RowSideColors= col1[as.numeric(group)]
)
# make a heatmap with a subsample of the final matrix (15x50)
heatmap.2(data.matrix(res.pca_filtro[,1:50]), col=greenred(75),
          trace="none",
          keysize=1,
          margins=c(6,20),
          scale="none",
          dendrogram="row",
          Colv = TRUE,
          Rowv = TRUE,
          cexRow=0.5 + 1/log10(dim(res.pca_filtro)[1]),
          cexCol=1.25,
          main="kmer-15 15x50",
          RowSideColors= col1[as.numeric(group)]
)
```

###

H-disregions analysis p pipeline

```
###GENERATE LIST OF K-MERS FREQUENCY###
AAF -> phylokmer.dat output

###FILTER KMERS WITH HIGH DISPERSION
KmerFilter.R -> Hdisp -> filter_kmer_list.fa

###EXTRACT READS WITH IDENTIFIED H-DISP REGIONS###
extract_kmers.pbs
cat ITV280_S1_R1_001.fastq | grep -B1 "AATCTCGTATGCCGTCTTCTGCTTG" >
3206_k1
```

```
cat ITV280_S1_R1_001.fastq | grep -B1 "AAGCAGAAGACGGCATACGAGATTC" >
3206_k2
cat ITV3206_S1_R1_001.fastq | grep -B1 "ACAAGTCTCGCTGTCTCTTGATCAC" >
3206_k3
cat ITV3206_S1_R1_001.fastq | grep -B1 "GATCAAGAGACAGCGAGACTTGTGA" >
3206_k4
...

###GENERATE THE FASTA FILE FOR THE H-DISP READS###
cat 3206_k1 3206_k2 3206_k3 ... > 3206_kmers.fasta
sed s'/@/> /g' 3206_kmers > 3206_kmers1
sed s'/--/ /g' 3206_kmers1 > 3206_kmers2
awk 'NF' 3206_kmers2 > 3206_kmers.fasta

###BLAST THE READS AGAINST NR###
blastx -num_threads 128 -db /bio/share_bio/softwares/blast_nr/tar_gz/nr -
query 3206_kmers.fasta -outfmt "6 qseqid  sseqid sgi stitle sstart send
mismatch pident qcovs evalue bitscore" -max_target_seqs 1 -evalue 1e-03 -
out 3206_kmers_blastx.tab

###SELECT GENES###
cat 3206_kmers_blastx.tab | cut -f 4,8,9 | grep "Ipomoea" | grep -v
"putative" | grep -v "probable" | grep -v "uncharacterized" >
3206_blast_Kmers_selected.tab

###GENE ASSEMBLY###
1.mapped to reference reads -> Bowtie2_proteins.pbs
bowtie2-build I_nil_ycf1.fa d I_nil_ycf1.fa
bowtie2 -p 64 -x d I_nil_ycf1.fa -1 ITV3206_S1_R1_001.fastq -2
ITV3206_S1_R2_001.fastq -S 3206_ycf1.sam
samtools view -b 3206_ycf1.sam > 3206_ycf1.bam
samtools sort -@ 64 -m 2G -o 3206_ycf1_sorted.bam 3206_ycf1.bam
samtools index 3206_ycf1_sorted.bam
samtools view -b -F 4 3206_ycf1_sorted.bam > 3206_ycf1_mapped.bam
samtools bam2fq 3206_ycf1_mapped.bam | seqtk seq -A > 3206_ycf1_mapped.fa

2. Gene assembly -> Geneious
      Map to Reference 1 -> contigs
      Bowtie remapping -> Bowtie2_pro_remap.pbs (ref: ycf1_contigs.fa)
      Map to reference 2 -> remapped
      Bowtie remapping Back -> Bowtie2_pro_remap_back.pbs (ref:
ycf1_cavalcantei_remap.fa)
      Gene annotation -> Augustus
```
